# Impact of capsaicin on aroma release and perception from flavoured solutions

**DOI:** 10.1016/j.lwt.2020.110613

**Published:** 2021-03

**Authors:** Ni Yang, Qian Yang, Jianshe Chen, Ian Fisk

**Affiliations:** a Division of Food, Nutrition and Dietetics, University of Nottingham, Sutton Bonington Campus, Loughborough, LE12 5RD, UK; b Lab of Food Oral Processing, School of Food Science and Biotechnology, Zhejiang Gongshang University, Hangzhou, China

**Keywords:** Chilli, Aroma release, Perception, Saliva, Individual differences

## Abstract

Capsaicin is the main component in chilli pepper, which contributes to the spiciness of the food. However, the role of capsaicin on aroma perception has been controversial in the literature. This is the first study exploring the impact of capsaicin on aroma release and perception simultaneously. Flavoured solutions with 3-methylbutanal (nutty note) were made with or without 5 mg/L capsaicin. Real-time APCI-MS analysis was applied to investigate in-nose aroma release during and after consumption of the solutions, and sensory tests were simultaneously conducted to reveal any temporal perception changes over 60 s. The results from 15 participants with triplicates indicated that capsaicin had no significant impact on aroma concentration from aqueous solutions, but the aroma perception rating was significantly higher (p < 0.0001), increasing by 45%. Capsaicin also enhanced average saliva flow by 92% (p < 0.0001), and lower saliva flow participants were found to have lower spiciness and aroma ratings.

## Highlights

• Capsaicin's impact on aroma release and simultaneous perception was investigated.
• Flavoured solutions of 3-methylbutanal were made with or without capsaicin.
• Capsaicin had no significant impact on the in-nose aroma release concentration.
• Capsaicin significantly enhanced aroma perception by 45% during 60s observation.
• Participants with lower saliva flow also had lower spiciness and aroma ratings.

## Graphical abstract

**Figure:**
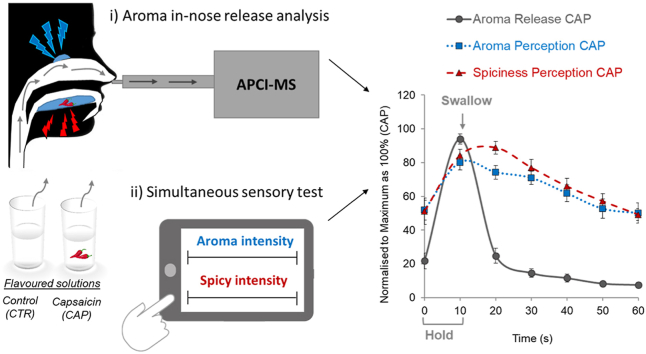

## 1 Introduction

Capsaicin (8-methyl-*N*-vanillyl-6-nonenamide) is one of the major capsaicinoids in chilli peppers, and it contributes to the pungent flavour or spiciness/piquancy of the food. Consumption of chilli pepper or capsaicin might offer a potential healthy eating strategy, as proposed by a recent review (Spencer & Dalton, 2020). Previous studies investigated the methods to extract capsaicinoids from chilli paste (Civan & Kumcuoglu, 2019), and non-targeted metabolomics analysis was recently applied to characterise and discriminate chilli peppers (Mi et al., 2020). However, the content of capsaicinoids varied from different chilli cultivars and processing methods (Loizzo, Pugliese, Bonesi, Menichini, & Tundis, 2015). Therefore, instead of using chilli pepper, this study used solutions made from pure capsaicin with known amounts.

Oral exposure of capsaicin triggers the trigeminal sensation in the oral cavity to activate TRPV1 (transient receptor potential vanilloid subtype 1) and elicit warm or burning sensations (Caterina et al., 1997; Yang & Zheng, 2017). The role of capsaicin as an oral chemical irritant on flavour perception has had controversial findings, although prior work primarily proposed a masking effect of capsaicin on gustatory and olfactory sensations (Lawless, Rozin, & Shenker, 1985). Prescott and Stevenson (1995) found that both flavour and sweetness ratings were suppressed by capsaicin in a complex taste and flavour mixture, but they failed to find a significant impact on flavour intensity when strawberry flavour presented alone.

The olfactory sensation linking with aroma perception is stimulated by volatile aroma compounds that release from food to the olfactory epithelium at the top of the nasal cavity otho-nasally or retro-nasally. Atmospheric pressure chemical ionisation-mass spectrometry (APCI-MS) is one of the real-time techniques to monitor aroma release, and its in-nose analysis measures aroma release are representative of those that interact with the receptors, which can be better correlated to sensory analysis. Previously, Davidson, Linforth, Hollowood, and Taylor (1999) combined this analytical approach with sensory Time-Intensity tests to evaluate the in-nose release of a minty compound and the mint perception during chewing gum consumption. However, this approach has never been applied to reveal the impact of oral capsaicin stimulation on aroma release and simultaneous sensory perception.

Smutzer et al. (2018) found that complete blockage of nasal airflow diminished capsaicin perception in the oral cavity, and they also reported that capsaicin perception intensity reduced by 75% and 50% after oral rinses with vanillin emulsion and sucrose solution respectively. They proposed a chemosensory link between receptor cells that detect sweet stimuli and trigeminal neurons that detect capsaicin detection. The cross-modal influences on the perception of spiciness were reviewed by Spence (2019), and he pointed out that our understanding about any possible multisensory interaction between capsaicin and basic tastes and aroma is very limited, so further research is needed to explore multisensory interactions with capsaicin.

On the other hand, a recent study (Gardner, So, & Carpenter, 2020) revealed that capsaicin stimulation significantly increased the abundance of most major salivary proteins (e.g., amylase and MUC7 as a mucin that is encoded by the MUC7 gene). In other studies (Pages-Helary, Andriot, Guichard, & Canon, 2014; Dinu et al., 2018), salivary proteins like mucin and α-amylase were found to affect flavour release by interacting with or metabolising flavour compounds. Therefore, we could propose that capsaicin induces various changes in saliva, which then affect the release of aroma compounds. This hypothesis was tested in our previous study (Yang, Galves, Racioni Goncalves, Chen, & Fisk, 2020). The results showed that capsaicin had little impact on aroma release in vitro, but it significantly decreased the in nose concentrations of all three aroma compounds. The reduction in the retronasal release observed might be due to the dilution and dissolution of these compounds by extra saliva stimulated by capsaicin.

The aim of this study was to explore the impact of oral capsaicin stimulation on in-nose aroma release and its simultaneous perception. We proposed two potential mechanisms relating to the impact of capsaicin on aroma perception. The first hypothesis is that capsaicin changes aroma perception by altering the level of aroma compounds releasing from the matrix to the nasal cavity due to the extra saliva stimulated by capsaicin, so less aroma compounds are released into the olfactory receptor and resulting in the reduced aroma perception. The second hypothesis is there is a multisensory interaction between aroma perception and trigeminal sensation triggered by capsaicin, and aroma perception might be enhanced/reduced due to its interaction with trigeminal sensation. Participants were asked to record their perceived spiciness and aroma intensities while breathing into the APCI-MS to measure their real-time aroma release. The inter-individual differences on saliva secretion and their consumption frequency of chilli food were also recorded, with the objectives of exploring any association with their aroma and spiciness perception. This is the first study designed to reveal the mechanism of capsaicin's effect on aroma perception combining aroma release analysis with simultaneous sensory tests.

## 2 Material and methods

### 2.1 Chemicals and sample preparation

Natural capsaicin was purchased from Sigma Aldrich, and 1% (1g/100g) stock solution was prepared by dissolving 0.1 g into 10 g ethanol (food-grade, 96%). One litre of the flavoured solution was made by adding 100 μL of 3-methylbutanal (food-grade, ≥ 97%, Sigma Aldrich) into pure water. Half of this solution (500 mL) was used as the control sample (CTR), and another 500 mL was made into capsaicin sample (CAP) by adding 250 μL of 1% capsaicin stock solution (final capsaicin concentration = 5 mg/L). The aqueous CTR and CAP solution were prepared one day before the experiment and stored in the Durian bottles with screw caps in the fridge (5 °C). The CTR and CAP samples were labelled with respective three-digit code. During the day of the experiment, these bottles were left at the room temperature 1 h before the static headspace analysis by APCI-MS, and then these samples were served at the room temperature to the participants in randomised orders.

### 2.2 Participant selection

Fifteen participants were recruited from the University of Nottingham (age 22-37) and the frequency of their spicy food consumption was recorded. A training session was provided to familiarise participants with testing protocols, detailed guidance and videos of instruction on how to conduct APCI-MS analysis and record the perceived flavour and spicy intensity simultaneously. Three separate sessions were arranged in the morning of three consecutive days to obtain three repeated measures. Each session included saliva collection, followed by synchronised APCI-MS and sensory analysis.

### 2.3 Saliva collection

A standard draining method (Bhattarai, Kim, & Chae, 2018) was used to collect saliva from 15 participants for 1min immediately after they swallowed a 10 mL solution (CTR or CAP sample). Samples were served in a randomised order, and every participant was asked to cleanse their palate with water before each saliva collection and take a break of at least 10 min between samples. After swallowing the sample, participants were instructed to keep their head tilted slightly forward and keep their mouth open to allow the saliva to drip passively from the lower lip into a sterilised cup for an entire minute. Participants were then asked to close the sterilised cup with a lid and pass it back to the researchers. Researchers weighed the container before and after the saliva collection. Saliva flow rate (g/m) was calculated using the weight difference (the amount of saliva secreted in grams) divided by time (1 min).

### 2.4 APCI-MS analysis

The MS Nose interface (Micromass, Manchester, UK) fitted to a Quattro Ultima mass spectrometer (Waters Corporation, Milford, MA) was used in this study. The selected ion mode was used with the cone voltage of 50 V, source temperature of 75 °C and a dwell time of 0.02 s. The transfer line temperature was set at 120 °C. The target ion for 3-methylbutanal is 87, and data were collected by Mass Lynx™ (Waters, UK).

The standard technique for APCI-MS analysis previously reported (Yang et al., 2020) was applied in this study for both in vitro and in vivo tests. The in vitro static headspace analysis was applied before in vivo analysis to evaluate any headspace concentration differences between CTR and CAP samples. Both types of samples (10 mL) were placed in 100 mL Duran bottles with screw caps, which were left at room temperature (20 °C) to equilibrate for 20 min. The static headspace of four replicates of CTR and CAP solutions were analysed in a randomised order.

During in vivo analysis, every participant took turns to conduct the tests with at least 15 min breaks between samples. Samples of CTR and CAP were served in a randomised order. Participants were asked to cleanse their mouth with water and then placed 10 mL of the solution in their mouth and breathed into the nose tube connected to the APCI-MS. They were asked to hold the solution for 10 s and breathe normally into the tube.

### 2.5 Synchronised sensory analysis

Sequential profiling (Methven et al., 2010) was used in this study to record the perceived intensity of spiciness and aroma intensity over 60 s at the same time that participants were performing the APCI analysis. At the same time as participants started to hold the sample in their mouth and breathe into the APCI tube, participants started to score their perceived aroma and spiciness intensity on a tablet with the temporal scales placed in front of them. Both sensory attributes were scored at the line scale with the left-end being the lowest intensity and right-end being the highest intensity. After swallowing, they kept scoring their perceived aroma and spicy intensity every 10 s while breathing into the APCI-MS tube till the 60 s ended. Data were collected every 10 s during and after consumption till 60 s, and intensities of spiciness and aroma perception were collected at each 10 s time point. Compusense at hand (Guelph, Canada) was used to collect the sensory data.

### 2.6 Data analysis

For in vitro static headspace analysis the maximum ion intensity (Imax) for each sample was recorded. Data from four replicates (CTR samples and CAP samples respectively) were analysed using *t*-test for each compound to determine if capsaicin significantly affected its static release (p < 0.05).

For in vivo breath-by-breath analysis, the area under the curve (AUC), and Imax were extracted from the chromatogram of the APCI-MS. The mean of Imax and AUC was calculated for every participant and the average of all the participants was calculated during every 10 s of the observing period. Then the maximum spiciness or aroma perception was recorded for individuals, and the respective average of CTR and CAP samples was calculated.

All the statistical data analysis was done by SPSS (IBM® SPSS® Statistics version 25) and XLSTAT Software ©-Pro (2020.1.3, Addinsoft, Inc). Multivariate analysis of variance (MANOVA) was applied to evaluate any significant differences between CTR and CAP samples (p < 0.05), and Tukey's post hoc test was conducted for each time point. Cluster analysis was performed by Agglomerative Hierarchical Clustering (AHC) by XLSTAT, and the classified groups from AHC were used to determine which variables are discriminative by Parallel Coordinates Plot using XLSTAT. Analysis of variance (ANOVA) with Tukey's post-hoc analysis was conducted to confirm the significant differences between the groups (p < 0.05).

## 3 Results

### 3.1 Impact of capsaicin on in-nose aroma release

A wide range of volatile compounds was detected from different cultivars of chilli (Korkmaz, Hayaloglu, & Atasoy, 2017). Three aroma compounds (3-methylbutanal, 1-octen-3-ol, linalool) were used in a previous study (Yang et al., 2020), and they were selected based on their –phyico-chemical properties (e.g., different hydrophobicities), so the resulting flavouring was not perceived as a typical familiar flavour (unlike a commercial strawberry flavouring for example), which was difficult for participants to define in terms of aroma perception. Therefore, a specific aroma compound (3-methylbutanal) was used in this study because this hydrophilic compound showed the largest reduction by capsaicin oral stimulation compared to other aroma compounds (Yang et al., 2020).

The aroma compound- 3-methylbutanal, also known as isovaleraldehyde, is a common aroma compound that can be found in various food, such as fresh ripened chilli (Korkmaz et al., 2017), cheese (Teter et al., 2020), coffee (Yang et al., 2016), and bread (Birch, Petersen, & Hansen, 2013). It was identified as one of the key odour active compounds in cocoa powder (Frauendorfer & Schieberle, 2006). When it was diluted in water at 100 mg/L in this study, it was perceived to have a cocoa-like odour and nutty flavour.

The in vitro release of 3-methylbutanal from the aqueous solution by APCI-MS static headspace analysis (Fig. S1) confirmed that there was no significant aroma release difference between CTR and CAP samples. This in vitro result was consistent with previously published findings (Yang et al., 2020). However, instead of an ice-cube system used in the initial study, a flavoured aqueous solution was used as the standard system in this study. This is because the aqueous solution could offer three major advantages: i) it was much easier to prepare; ii) it could be kept at room temperature to serve to all the participants, iii) it has a short consumption period and would not change the temperature of the mouth drastically compared to the ice-cube system. To produce representative results using an aqueous solution, a standardised consumption protocol was established by asking every participant to hold the solution in the mouth for 10 s with minimum mouth movement, whilst breathing normally into the APCI-MS. This 10 s holding time also allowed participants to score their aroma and spiciness perception before swallowing the solution.

**Fig. 1:**
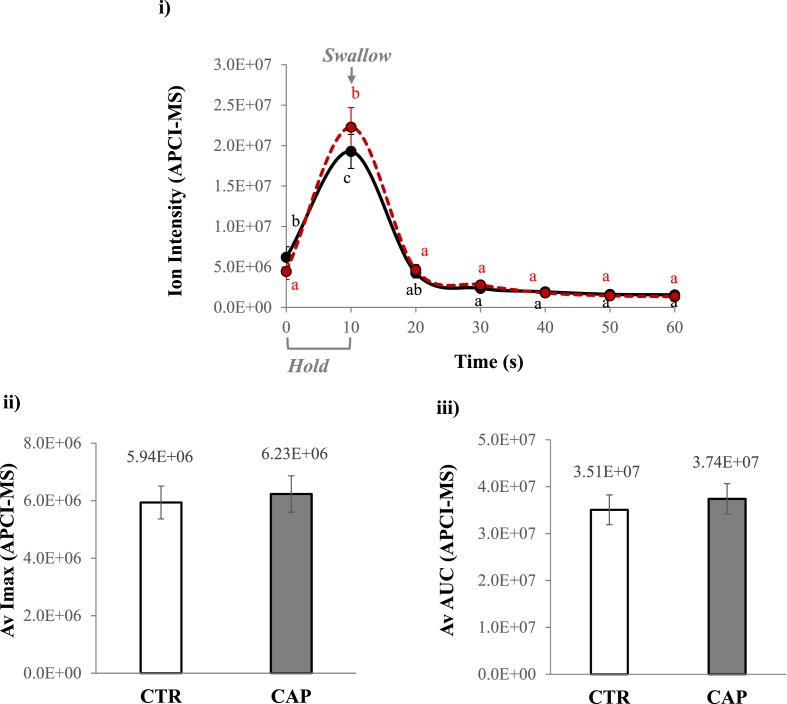
**Impact of capsaicin on in-nose aroma release**. i) Aroma release intensity from APCI-MS analysis over 60 s for CTR (black solid line with the dot) and CAP (red dash line with the dot); different letters (a-c) indicated significant differences between time points (p < 0.05). Average data of ii) Imax and iii) AUC for CTR (white bar) and CAP (grey bar); no statistical difference between CTR and CAP (p > 0.05). Data were based on the average of 15 participants x 3 reps for each sample. The standard error is shown as ± error bar. (For interpretation of the references to colour in this figure legend, the reader is referred to the Web version of this article.)

For the current in vivo study with 15 participants with triplicates, the dynamic changes of aroma release in the nose are demonstrated in Fig. 1 i). Either CTR or CAP solution was held in the mouth for 10 s, and aroma release reached its maximum immediately after swallowing at 10 s, then no significant differences after 20 s till 60 s. Statistically, the average of Imax and AUC results (Fig. 1 ii) and iii)) showed no significant difference between CTR and CAP (p > 0.05), so capsaicin did not affect the in-nose concentration from the aqueous solution. This finding seemed to reject the first hypothesis of this study that aroma release might be reduced with oral capsaicin stimulation. The in nose results from simple aqueous solutions in this study were different from our previous results using ice-cube systems, where embedded capsaicin caused a significant reduction on the level of aroma released (Yang et al., 2020). The reason could be because aroma release was extended over 1 min when the ice cube gradually melted in the mouth, compared to a quick release from simply drinking the aqueous solution, making it challenging to observe a significant capsaicin effect on the amount of volatiles released.

### 3.2 Impact of capsaicin on temporal sensory perception

**Fig. 2:**
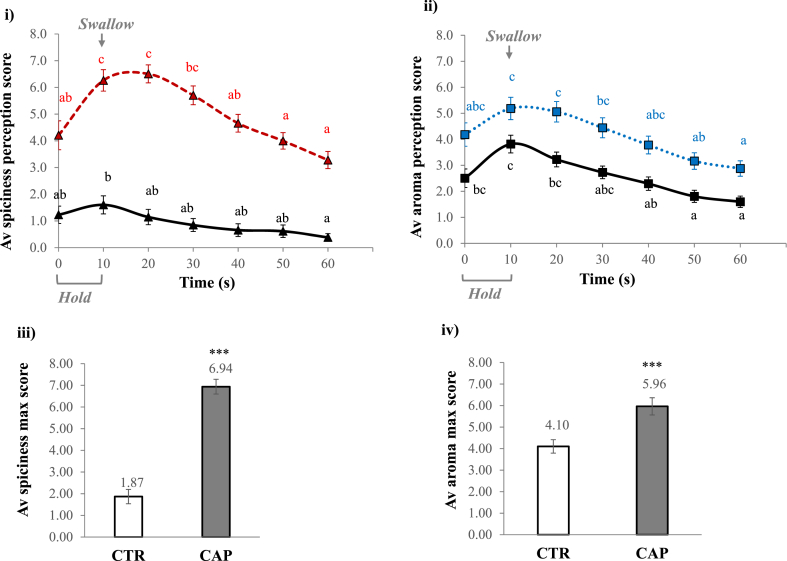
**Impact of capsaicin on temporal aroma perception.** i) spiciness perception scores over 60 s for CTR (black solid line with the triangle) and CAP (red dashed line with the triangle) and ii) aroma perception scores over time for CTR (black solid line with the square) and CAP (blue dotted line with the square) when solutions were held in the mouth for 10 s and swallowed after 10 s till 60 s. Different letters (a-c) indicated significant differences between time points (p < 0.05). Average data of iii) spiciness maximum score and iv) aroma maximum score for CTR (white bar) and CAP(grey bar); *** indicated a statistical difference of p < 0.0001. Data were based on the average of 15 participants x 3 reps for each sample. The standard error is shown as ± error bar. (For interpretation of the references to colour in this figure legend, the reader is referred to the Web version of this article.)

For the simultaneous sensory analysis, Fig. 2 i) and ii) display the results of spiciness and aroma perception scores at every 10 s until 60 s. Regarding spiciness perception, CAP (red dashed line) had much higher ratings than CTR (black solid line) throughout this 60 s period, and the maximum intensity of CAP was perceived after swallowing. The spiciness scores for CTR were around 1-2, which could be the baseline or noise from the panellists as no capsaicin was added. The average maximum spiciness (Fig. 2 iii) for CAP was more than x 5 significantly higher than CTR (p < 0.001). In Fig. 2 ii). aroma perception scores were also consistently higher for CAP than CTR during the 60 s observation period. The average maximum aroma perception (Fig. 2 iv) was significantly higher for CAP than CTR (p < 0.001), and rated aroma intensity from CTR (4.10) increased by 45% when comparing to CAP's average score (5.96). The enhanced aroma perception by capsaicin might provide a clue for the multisensory interaction proposed as the second hypothesis in this study.

The finding of capsaicin-enhanced aroma perception in this study was not reported in previous studies (Green, 1996; Lawless et al., 1985; Prescott & Stevenson, 1995). In contrast, they reported that capsaicin caused a reduction in flavour perception. However, the flavourings they used were more complex flavourings that were more associated with sweet flavours. For example, peach, pear, apricot and passion fruit flavour were investigated by Lawless et al. (1985); strawberry, vanilla and orange flavouring were investigated by Prescott and Stevenson (1995). These findings could be linked to the incongruent association and previous experiences (Frasnelli, Oehrn, & Jones-Gotman, 2009), because spicy is commonly associated with savoury flavours rather than sweet flavours. In our study, 3-methylbutanal with nutty flavour might offer a more neutral perception, so it is envisaged that more data on more congruent flavour (such as savoury) in the future will consolidate the knowledge on capsaicin's effect on aroma perception.

### 3.3 Impact of capsaicin with a combination of analytical and sensory data

**Fig. 3:**
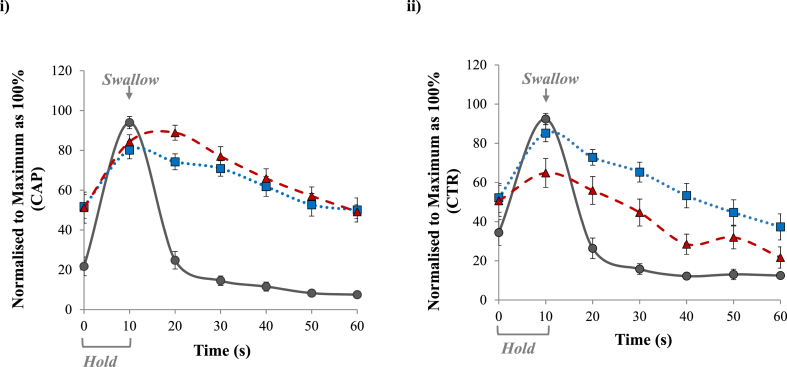
**Impact of capsaicin with a combination of analytical and sensory data.** Data were normalised to the individual's maximum values as 100%, and the results showed in the figure were the average of 15 subjects. i) capsaicin solution (CAP) and ii) control solution (CAP). Normalised aroma release (solid grey line with the dots), aroma perception (blue dotted line with squares) and spiciness perception (red dashed line with the triangle) during 60 s observation period. (For interpretation of the references to colour in this figure legend, the reader is referred to the Web version of this article.)

In this study, data from aroma release was normalised to the individual's respective maximum values as 100%, and the results were the average of 15 subjects. The same approach was applied to aroma perception and spiciness perception. As illustrated in Fig. 3 i) for normalised CAP results, in-nose aroma release reached its 100% at 10 s and quickly declined, but aroma perception (blue dotted line) did not show similar patterns as the aroma release profile (grey solid line), but its curve followed a very similar shape as the spiciness perception (red dashed line). However, a similar pattern was observed for normalised CTR data in Fig. 3 ii), where aroma perception did not follow aroma release, but had a more similar trend to spiciness perception. This might be due to a potential “Halo” effect when two attributes (spiciness and aroma perception) were scored at the same time. Although clear instructions were given to the participants and a trial session was conducted, they might still be confused about aroma perception with spiciness perception. To confirm the aroma-capsaicin multisensory interaction, further studies on fully trained participants would be essential to evaluate whether they would give any different results.

Additionally, another possible explanation is that the consumption of capsaicin may reduce the threshold of aroma perception, which leads to a much higher intensity of aroma perception, despite no significant change in aroma concentration released in the nose. Lv et al. (2020) discovered that viscosity discrimination threshold decreased after rinsing the mouth with capsaicin (20 mg/L), and tongue temperature was increased by 1.3 °C. Although this increase was not large, its effect was a long-lasting during the 60 s observation period. The effect of temperature was reported with a significant influence on taste intensity, detection and recognition threshold (Green, Alvarado, Andrew, & Nachtigal, 2016; Green & Nachtigal, 2015). However, limited literature was found about the thermal impact on the aroma perception threshold, so it will be useful to examine whether capsaicin consumption that elevates tongue temperature would affect aroma perception threshold retronasally in future research.

### 3.4 Impact of capsaicin on saliva secretion

**Fig. 4:**
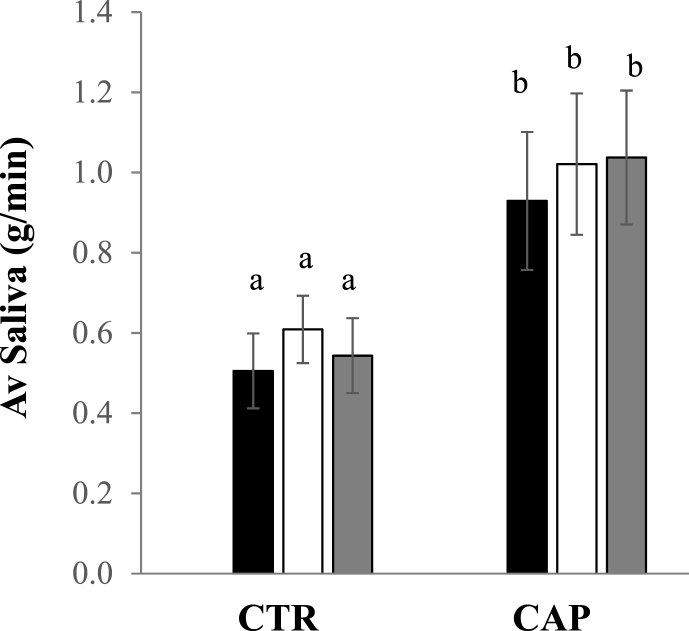
**Impact of capsaicin on saliva secretion**Average saliva flow (g/min) measured from 15 participants for CTR and CAP on three consecutive days (rep 1,2, 3 shown in black, white, grey bars with standard errors), letter a and b indicated a statistical difference at p < 0.001.

A standard saliva collection method was applied for 15 participants when they held either 10 mL CTR or CAP solution in the mouth for 10 s, and saliva was collected for 60 s after swallowing the solution. This resembled the same procedure used for APCI-MS and sensory analysis. The average saliva flow of all the participants calculated from three consecutive days (rep 1, 2, 3) are shown in Fig. 4 i). Statistically, saliva flow during 60 s after swallowing of CAP solution had a significantly higher rate than its flow rate after CTR consumption (p < 0.001), and no significant difference was found between replicates collected on different days. The average saliva flow for CTR was calculated as 0.55 g/min (Table 1). It increased to 1.00 g/min after capsaicin stimulation, which indicated that nearly double the amount of saliva was secreted.

**Table 1:** Average saliva flow (g/min, ± STD) after consumption of CTR and CAP solution, saliva flow ratio of CAP/CTR and chilli food consumption frequency per week recorded for individual participants (P1-15). Average (AV) and standard deviation (STD) of all the participants were also calculated.

Participants	CTR saliva flow (g/min)	CAP saliva flow (g/min)	Saliva flow ratio (CAP/CTR)	Frequency of chilli food consumption
P1	1.30 ± 0.11	1.03 ± 0.33	0.79	3.5
P2	0.59 ± 0.24	1.03 ± 0.53	1.75	7.0
P3	0.31 ± 0.13	0.56 ± 0.30	1.82	5.0
P4	0.46 ± 0.18	0.72 ± 0.08	1.56	6.0
P5	0.11 ± 0.04	0.24 ± 0.06	2.17	0.0
P6	0.56 ± 0.11	1.46 ± 0.62	2.61	2.0
P7	0.52 ± 0.23	0.82 ± 0.07	1.58	7.0
P8	0.29 ± 0.05	0.54 ± 0.20	1.88	3.5
P9	0.61 ± 0.06	1.70 ± 0.18	2.80	1.5
P10	0.58 ± 0.37	0.71 ± 0.07	1.21	1.0
P11	0.48 ± 0.26	1.32 ± 0.36	2.77	7.0
P12	0.50 ± 0.23	1.88 ± 0.99	3.74	4.0
P13	0.49 ± 0.24	0.47 ± 0.13	0.95	3.0
P14	1.20 ± 0.10	2.04 ± 0.75	1.70	3.5
P15	0.30 ± 0.07	0.42 ± 0.05	1.42	3.0
AV	0.55 ± 0.31	1.00 ± 0.55	1.92 ± 0.76	

Generally, this study observed that an average of an extra 92% saliva was stimulated after swallowing 5 mg/L capsaicin solution (Table 1). The earlier study (Yang et al., 2020) reported that an extra 75% saliva was stimulated by placing the cotton immersed with 5 mg/L capsaicin in the mouth. Although these two studies used different collection methods with different numbers of participants, both findings confirmed a significant enhancement of saliva secretion by oral exposure to capsaicin. These extra amounts of saliva played a significant role in aroma release from the ice-cube system, but it might have limited impact from aqueous solution as volatile aroma compounds were quickly exhaled and disappeared after swallowing. However, if a more complicated food system (like solid matrix) is considered, the role of extra saliva stimulated by capsaicin on aroma release and taste release should not be underestimated.

### 3.5 Individual differences

In the absence of capsaicin stimulation, individual subject's (P1-P15) average saliva flow rate (Table 1) showed large variations among 15 participants (ranging from 0.11 to 1.30 g/min), which indicated more than ×10 difference in their innate saliva secretion capability. Comparing CAP to CTR as the saliva ratios of CAP/CTR, 13 out of 15 participants had a ratio higher than 1.0, indicating they had higher saliva flow after oral exposure of capsaicin. The average CAP/CTR ratio is 1.92, indicating that additional 92% of saliva generated by capsaicin in average. However, the CAP/CTR ratio was less than 1.0 for two participants (0.79 for participant P1, and 0.95 for participant P13), meaning capsaicin might have a different impact on their saliva generation. Potentially, chilli consumption frequency could have a significant effect on saliva secretion, so this data is included in Table 1. However, these two subjects (P1 and P13) indicated a medium frequency for chilli consumption (3.5 and 3.0 times per week, respectively), so the frequency of chilli consumption did not explain their saliva results, and no significant correlation was found between their saliva secretion and frequency of chilli food consumption using Pearson's correlation (p > 0.05).

**Fig. 5:**
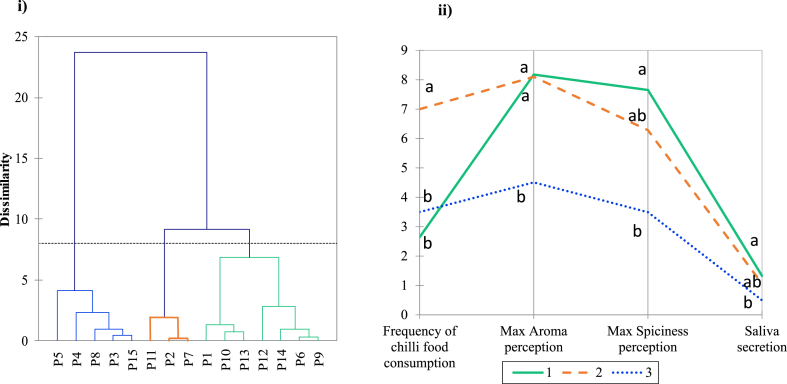
**Impact of capsaicin with individual differences**. i) Dendrogram of all 15 participants for CAP solutions and ii) plots of three groups of participants (Group 1, green solid line; Group 2, orange dashed line; Group 3, blue dotted line) based on how often eat spicy food a week, maximum aroma perception, maximum spiciness perception and saliva secretion for CAP solutions. Letter a and b indicated a significant difference between the groups for each factor by Tukey's post-hoc analysis (p < 0.05). (For interpretation of the references to colour in this figure legend, the reader is referred to the Web version of this article.)

To explore if any participants could be classified according to particular factors, cluster analysis was conducted based on their frequency of chilli consumption, maximum aroma perception, maximum spiciness perception and saliva secretion for CAP samples. The resulting Dendrogram and parallel coordinates plot map are shown in Fig. 5 i) and ii) respectively. Three groups of participants were identified, including Group 1 (green lines) with 7 subjects, Group 2 (orange lines) with 3 subjects, and Group 3 (blue lines) with 5 subjects. Group 1 subjects had the lowest frequency of chilli consumption (less than 3 times a week) and scored the highest maximum spiciness and aroma perception, and they also generated the highest level of saliva compared to the other two groups. It is rational to suggest that these participants with less capsaicin consumption experience were more likely to generate more saliva and had higher perception scores. Comparing with Group 1, Group 2 subjects had the highest chilli consumption frequency (7 times per week) and also scored high on aroma perception, but they had lower spiciness scores with a lower level of saliva stimulated by capsaicin. This is likely due to them adapting to capsaicin stimulation and generating less saliva with lower spiciness perceived. Interestingly, Group 3 subjects chilli consumption frequency ~3.5 times a week on average, not significantly different from Group 2) had the lowest spiciness and aroma perception with the lowest saliva secretion. Compared to the chilli consumption frequency, the saliva level stimulated by capsaicin seemed to have a stronger association with the maximum aroma and spiciness perception.

Linking to other studies, Lawless et al. (1985) found that non-eaters had higher burning ratings than eaters did, and Prescott and Stevenson (1995) had the same finding. Parts of our results are in agreement with these two studies: Group 1 subjects with lowest chilli consumption frequency (<3 times a week) rated the highest spiciness, and Group 2 subjects with the highest chilli consumption frequency (7 times a week) had lower spiciness scores than Group 1. However, the results for Group 3 subjects cannot be explained in the same way, as these subjects had medium consumption frequency (~3.5 times a week) close to Group 1, but they rated the lowest spiciness intensity. The possible interpretation is that compared to chilli consumption frequency, the level of saliva stimulated by capsaicin is a more important factor. Participants from Group 3 with the lowest saliva secreted by capsaicin were more likely to be affected by capsaicin with much lower aroma intensity perceived.

In general, the proposed mechanism might be that capsaicin enhances saliva flow and elicits spiciness, and spiciness enhances aroma perception through multisensory interaction. However, this capsaicin-aroma multisensory interaction linking with our second hypothesis will require further validation, for example, using functional magnetic resonance imaging (fMRI) to consolidate this interaction at the brain level in future. The database in this study might be too small to compare with larger scales of consumer studies, but it might provide a useful insight to the food manufacturers that designing personalised food products could be beneficial for different consumer groups.

## 4 Discussion

One unique design of this study was to monitor the impact of capsaicin on aroma release and simultaneous perception during and after consumption. Despite no significant difference observed for in-nose aroma concentration from aqueous systems, sensory results revealed that aroma perception was enhanced by 45% in CAP compared to CTR. A future study could examine if CAP solution with 45% less aroma added would result in similar aroma intensity perception as CTR with 100% aroma. Food companies that apply spicy ingredients to their original formulation could examine if capsaicin might offer a cost-effective benefit with fewer flavourings required and yet the participants’ aroma perception remaining the same. However, this finding was based on one aroma compound (3-methylbutanal), and further validation is required when complex flavourings are used. Particularly, different aroma compounds with distinct physicochemical properties might interact with capsaicin-saliva matrix in different ways, so we would like to propose future studies on exploring the mechanism of these interactions with different aroma compounds.

The results of this study were based on 5 mg/L capsaicin, which was equivalent to 80 SHU - Scoville Heat Units (Todd, Bensinger, & Biftu, 1977). Previously, Prescott and Stevenson (1995) verified that different levels of capsaicin (1, 4, 16 mg/L) affected the differences in flavour perception between frequent and infrequent chilli users. So it would be beneficial for future studies to conduct an additional evaluation on different levels of capsaicin. Particularly, using lower levels of capsaicin (like 2 mg/L) in future studies might minimise the overwhelming spiciness sensation that is likely to induce the “Halo” effect when aroma perception is also simultaneously judged.

Besides the aqueous systems used in this study, future studies could focus on dry matrices to evaluate the impact of capsaicin, because longer oral processing time will be involved for chewing the solid matrix, and saliva will play a vital role to hydrate and release flavour compounds from these matrices. However, individual differences in their oral physiology should be evaluated when more complex foods are consumed. For example, Gierczynski, Laboure, and Guichard (2008) demonstrated that some physiological parameters (like chewing activity, frequency of velum opening, mouth-coating) were used to differentiate three groups of subjects according to their release behaviour. Moreover, increasing the fat level was known to reduce the perceived spiciness (Baron & Penfield, 1996). Blending specific ingredients like fat, starch and sugar in the food products also showed a significant impact on sensory rating and the total capsaicinoids concentration (Schneider, Seuß-Baum, & Schlich, 2014). Hence, other ingredients in the food should also be taken into account, because they were known to interact with capsaicin and flavour compounds.

The breakthrough of this study was to illustrate the significant correlation of saliva flow with spiciness and aroma perception. A greater amount of saliva stimulated by capsaicin was more likely to have higher spiciness and aroma perception scores (Fig. 5 ii). Although Nasrawi and Pangborn (1990) did not find a significant correlation between parotid salivary response and perceived sensory responses due to large inter-subject variability, they noticed that 9 subjects with high innate saliva flow were also more affected by capsaicin with increased saliva, compared to 14 low flow subjects. In this study, the cluster analysis also identified different groups of participants: the highest saliva flow subjects generally had the highest aroma and spiciness scores; and the lower saliva flow subjects had the lowest aroma and spiciness intensities. However, 15 participants is too small a sample size to conclude, so it will be useful to involve a larger number of participants (e.g., 100, 200 and above) to confirm whether different groups of behaviour could be observed.

Additionally, the findings in this study also indicated that the innate saliva flow among 15 participants could have more than ×10 differences, without any capsaicin stimulation. As pointed out by Canon, Neiers, and Guichard (2018), saliva is a key factor for flavour release and perception due to its diverse functions, such as controlling the transport of flavour molecules to their receptors, adsorption to the oral mucosa, enzymatic metabolism orally, etc. They also reported that the great inter-individual variation in salivary composition was associated with different flavour perception. Therefore, variability in saliva generation, salivary composition and salivary proteins among individuals might also influence how capsaicin-saliva matrix could affect the release of aroma compound in this study.

## 5 Conclusions

In conclusion, the combination of analytical and sensory analysis in this study provided a unique understanding of the impact of capsaicin on real-time aroma release and its immediate perception. In simple aqueous systems of 3-methylbutanal (nutty note) with 5 mg/L capsaicin, the average in-nose release was not significantly affected by oral capsaicin stimulation, but aroma perception was significantly increased by 45%. These findings disproved the first hypothesis that capsaicin might have an indirect impact on aroma perception by stimulating extra saliva to cause a reduction on aroma compounds available to the receptors in the nose, and hence resulting in reduced aroma perception. On the other hand, the results of synchronised aroma perception did not match the dynamic in-nose aroma release yet followed a similar pattern of spiciness perception over time, but the control system also showed similar results, which indicated that there might be the “Halo” effect when the participants rated their perceived aroma intensity and spiciness intensity at the same time. Therefore, further investigations are required to confirm the second hypothesis on the nature of capsaicin-aroma multisensory interaction.

Additionally, capsaicin largely increased saliva flow, by 92% on average, but a large inter-individual difference was observed with more than 10 times difference on their innate saliva flow rate. Compared to the chilli consumption frequency, participants were more likely to be grouped according to their capsaicin-stimulated saliva flow and association with their perceived aroma and spiciness maximum intensity. With oral exposure to capsaicin stimulation, subjects with a lower level of saliva stimulation scored lower in their spiciness and aroma perception, compared to subjects with a higher amount of saliva stimulated by capsaicin.

## Ethical approval

Ethics approval (SBREC170129A) was obtained from the School of Biosciences at the University of Nottingham, and all test procedures followed the ethical rules and regulations set by the University.

## CRediT authorship contribution statement

**Ni Yang:** Conceptualization, Data curation, Formal analysis, Funding acquisition, Investigation, Methodology, Project administration, Resources, Software, Supervision, Validation, Visualization, Writing - original draft, Writing - review & editing. **Qian Yang:** Conceptualization, Formal analysis, Investigation, Methodology, Supervision, Software, Writing - original draft, Writing - review & editing. **Jianshe Chen:** Funding acquisition, Conceptualization, Formal analysis, Writing - original draft, Writing - review & editing. **Ian Fisk:** Funding acquisition, Conceptualization, Formal analysis, Writing - original draft, Writing - review & editing.

## Declaration of competing interest

The authors declare that they have no competing financial interests.

## Appendix A Supplementary data

Fig. S1 Average Imax of 3-methyl butanal in vitro release by APCI-MS static headspace analysis for CTR (white bar) and CTR (grey bar). The standard error is shown as ± error bar, and no statistical difference by ANOVA analysis (p > 0.05). Data were based on the average of 4 reps for each sample measured for 3 days of the experiment. Fig. S1
