# Supplementary figures and images for: Impact of capsaicin on aroma release and perception from flavoured solutions
Source: Lebensm Wiss Technol. 2021 Mar;138:110613. doi: 10.1016/j.lwt.2020.110613 (PMC7829613; doi:10.1016/j.lwt.2020.110613)

## Slide 1
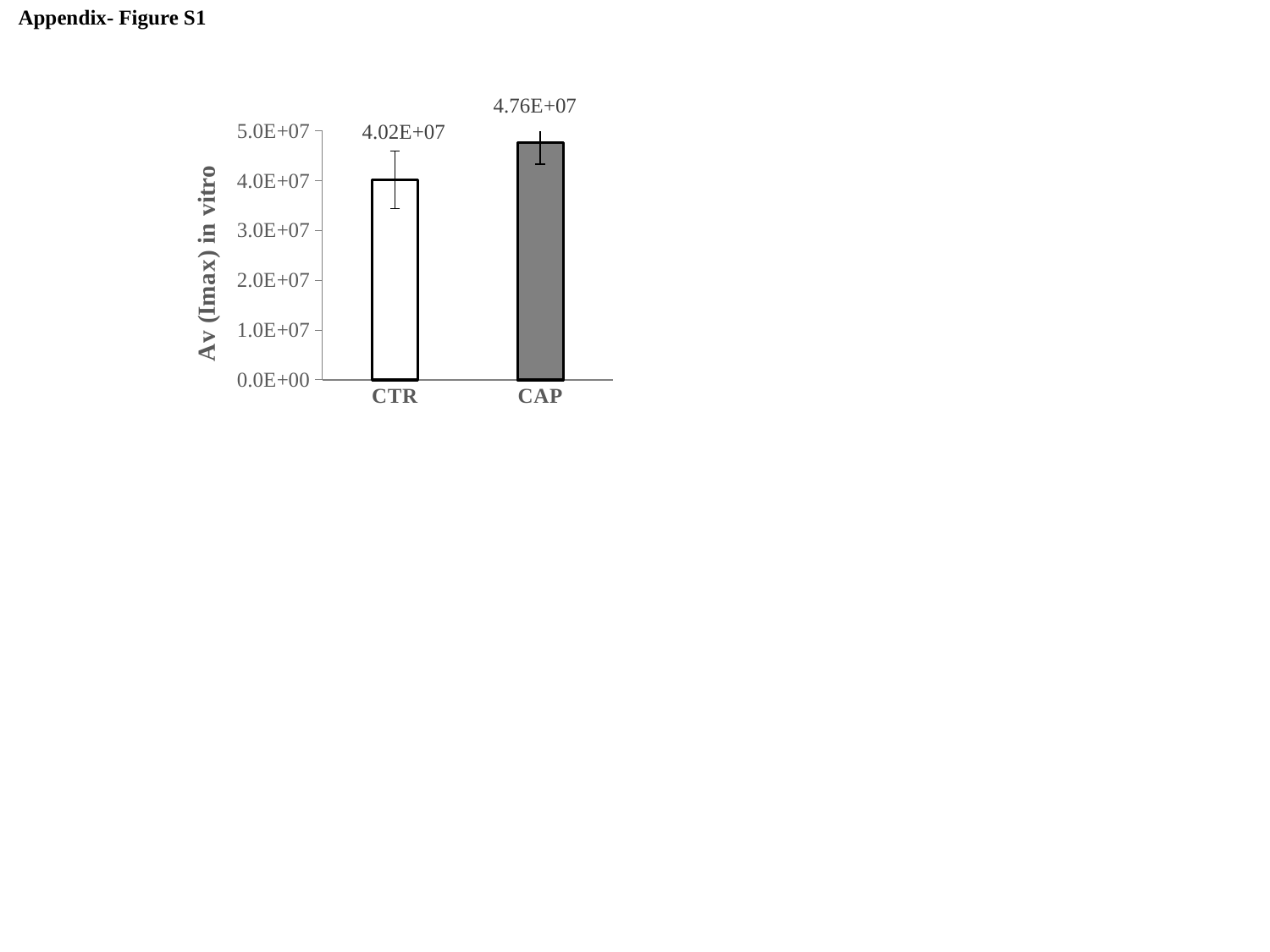

Appendix- Figure S1
### Chart
| Category | |
|---|---|
| CTR | 40166666.666666664 |
| CAP | 47600000.0 |

Supplement: Fig. S1 — Average Imax of 3-methyl butanal in vitro release by APCI-MS static headspace analysis for CTR (white bar) and CTR (grey bar). The standard error is shown as ± error bar, and no statistical difference by ANOVA analysis (p > 0.05). Data were based on the average of 4 reps for each sample measured for 3 days of the experiment. [file mmc1.pptx]
